# Type 2 Diabetes Mellitus and Latent Tuberculosis Infection Moderately Influence Innate Lymphoid Cell Immune Responses in Uganda

**DOI:** 10.3389/fimmu.2021.716819

**Published:** 2021-08-27

**Authors:** Phillip Ssekamatte, Marjorie Nakibuule, Rose Nabatanzi, Moses Egesa, Carol Musubika, Mudarshiru Bbuye, Matthew R. Hepworth, Derek G. Doherty, Stephen Cose, Irene Andia Biraro

**Affiliations:** ^1^Department of Immunology and Molecular Biology, School of Biomedical Sciences, Makerere University College of Health Sciences, Kampala, Uganda; ^2^Immunomodulation and Vaccines Programme, Medical Research Council/Uganda Virus Research Institute (MRC/UVRI) and London School of Hygiene and Tropical Medicine (LSHTM) Uganda Research Unit, Entebbe, Uganda; ^3^Department of Infection Biology, Faculty of Infectious and Tropical Diseases, LSHTM, London, United Kingdom; ^4^Division of Infection, Immunity & Respiratory Medicine, School of Biological Sciences, Faculty of Biology, Medicine and Health, Lydia Becker Institute of Immunology and Inflammation and Manchester Collaborative Centre for Inflammation Research (MCCIR), Manchester, United Kingdom; ^5^Department of Immunology, Trinity College, Dublin, Ireland; ^6^Department of Internal Medicine, School of Medicine, Makerere University College of Health Sciences, Kampala, Uganda

**Keywords:** innate lymphoid cells, type 2 diabetes mellitus, tuberculosis, cytokines, hyperglycaemia, HbA1c

## Abstract

**Background:**

Type 2 diabetes mellitus (T2DM) is a major risk factor for the acquisition of latent tuberculosis (TB) infection (LTBI) and development of active tuberculosis (ATB), although the immunological basis for this susceptibility remains poorly characterised. Innate lymphoid cells (ILCs) immune responses to TB infection in T2DM comorbidity is anticipated to be reduced. We compared ILC responses (frequency and cytokine production) among adult patients with LTBI and T2DM to patients (13) with LTBI only (14), T2DM only (10) and healthy controls (11).

**Methods:**

Using flow cytometry, ILC phenotypes were categorised based on (Lin^−^CD127^+^CD161^+^) markers into three types: ILC1 (Lin^−^CD127^+^CD161^+^CRTH2^-^CD117^−^); ILC2 (Lin^−^CD127^+^CD161^+^CRTH2^+^) and ILC3 (Lin^−^CD127^+^CD161^+^CRTH2^−^NKp44^+/−^CD117^+^). ILC responses were determined using cytokine production by measuring percentage expression of interferon-gamma (IFN-γ) for ILC1, interleukin (IL)-13 for ILC2, and IL-22 for ILC3. Glycaemic control among T2DM patients was measured using glycated haemoglobin (HbA1c) levels. Data were analysed using FlowJo version 10.7.1, and GraphPad Prism version 8.3.

**Results:**

Compared to healthy controls, patients with LTBI and T2DM had reduced frequencies of ILC2 and ILC3 respectively (median (IQR): 0.01 (0.005-0.04) and 0.002 (IQR; 0.002-0.007) and not ILC1 (0.04 (0.02-0.09) as expected. They also had increased production of IFN-γ [median (IQR): 17.1 (5.6-24.9)], but decreased production of IL-13 [19.6 (12.3-35.1)]. We however found that patients with T2DM had lower ILC cytokine responses in general but more marked for IL-22 production (median (IQR): IFN-γ 9.3 (4.8-22.6); IL-13 22.2 (14.7-39.7); IL-22 0.7 (IQR; 0.1-2.1) p-value 0.02), which highlights the immune suppression status of T2DM. We also found that poor glycaemic control altered ILC immune responses.

**Conclusion:**

This study demonstrates that LTBI and T2DM, and T2DM were associated with slight alterations of ILC immune responses. Poor T2DM control also slightly altered these ILC immune responses. Further studies are required to assess if these responses recover after treatment of either TB or T2DM.

## Introduction

Tuberculosis (TB) still remains the leading cause of death from a single infectious agent globally despite advances in care in the past decade (1). One-third of the population globally is infected with the bacteria, *Mycobacterium tuberculosis* (*Mtb*) that causes the disease, and an estimated 10% of these are likely to develop active tuberculosis in their lifetime (1). Comorbidities such as Type 2 diabetes mellitus (T2DM) are known to influence the outcome of *Mtb* infection (1). T2DM patients are more susceptible to bacterial pathogens including *Mtb* (2, 3). T2DM confers a 3-fold increase in the risk of developing active TB in latent tuberculosis-infected individuals (4). It is associated with poor TB treatment outcomes including failure, relapse and death (4). The susceptibility to *Mtb* in T2DM patients is attributable to several factors including the direct effects of hyperglycaemia and consequently insulin resistance, and indirectly largely attributable to the immune-compromised state in this population (5). Both innate and adaptive immune responses are dysregulated in people having T2DM, which delays *Mtb* clearance (6–9).

Innate lymphoid cells (ILCs) are a novel family of hematopoietic effectors that play protective functions in innate immune responses to microorganisms, in lymphoid tissue formation, in tissue remodelling after the damage inflicted by injury and homeostasis of stromal tissue cells (10). Innate lymphoid cells are tissue-resident cells mainly found at mucosal surfaces of the intestine (11, 12), lungs (13) and in the skin (14), making them among the first immune cells to react to pathogens (15). There are three types of ILCs; ILC1s, ILC2s and ILC3s (16, 17), that have effector-immune functions and are involved in adipose tissue metabolism (18, 19). ILCs, unlike adaptive lymphocytes, do not express known rearranged antigen receptors but do exhibit T-cell-like functional diversity. Hence, ILC1s, ILC2s and ILC3s are innate counterparts of the T_H_1 and T_H_2 and T_H_17 subsets (respectively) of helper T cells in the adaptive immune system (17, 20). ILC1 cells are comprised of conventional natural killer cells and *bona fide* non-NK ILC1 (21). They respond to interleukin (IL)-12, IL-15 and IL-18 and are defined by their expression of the transcription factor T-bet (for function and development), and production of a characteristic type 1 effector cytokine profile, including interferon-gamma (IFN-γ), tumour necrosis factor (TNF) and oxygen radicals (17, 21–23). IFN-γ is a potent macrophage activator, enhances the phagocytic activity of the macrophages, and is one of the key cytokines conferring protection against TB (23–25). Type 2 ILCs respond to tissue-derived alarmins including IL-25 and IL-33 and thymic stromal lymphopoietin (TSLP) (14). They are characterized by their expression of GATA-3 and produce the cytokines IL-4, IL-5, IL-9, and IL-13 (13, 26–28) to combat helminth infection (29), mediate tissue repair (13, 15, 17, 30) and play roles in thermogenesis (19). Blood ILC1 & ILC2 have been implicated in TB infection (31). Type 3 ILCs respond to IL-23 and IL-1β, are characterised by their expression of RORγt and produce cytokines IL-17A and/or IL-22 (32, 33). They mediate resistance to intestinal infection (12, 34), and control early-stage TB infection (31). The three groups of ILCs have been implicated in adipose obesity-induced diabetes (35–38).

We hypothesized that since T2DM is characterised by an immune-suppressive state, the frequency of ILC subsets and their secreted cytokines would also be diminished.

## Materials and Methods

### Participants

Latent TB infection and/or T2DM participants were enrolled from October 2017 to March 2018 in the Tuberculosis and Diabetes (TAD) study, a case-control study assessing the frequency of ILCs, T and B cells in LTBI and/or T2DM patients. T2DM patients were newly diagnosed, hence they were not on DM treatment or TB preventive therapy that would affect immune function. All participants were enrolled after providing informed consent and approval by the institutional review boards including the Higher Degrees Research and Ethics Committee (HDREC) of the School of Biomedical Sciences, Makerere University (Reference number; SBS-531) and Uganda National Council for Science and Technology (Reference number; HS66ES). All data were anonymised. For this study, stored peripheral blood mononuclear cell (PBMC) samples taken 48 from participants were used, of which 13 were LTBI/T2DM, 14 were T2DM only, 10 were LTBI only, and 11 were healthy controls (HC). T2DM was diagnosed using the American Diabetes Association (ADA) criteria based on glycated haemoglobin (HbA1c) levels ≥ 6.5% (39) and latent TB infection based on positive results for the immunological test QuantiFERON TB-Gold-Plus (QFT). All participants were HIV negative.

### PBMC Isolation

PBMCs were isolated from participants by density centrifugation using a Ficoll gradient. Cells were resuspended in cold heat-inactivated foetal bovine serum (FBS) supplemented with 10% dimethyl sulfoxide (DMSO), transferred to liquid nitrogen for long term storage following an overnight incubation in a controlled freezing container (Mr Frosty™, Nalgene).

### PBMC Stimulation and Culture

Frozen PBMCs were thawed in R10 (RPMI with 10% FBS, 1% Pen/Strep, 2 mM Glutamine, 25 mM HEPES) at 37°C and counted manually using a microscope. PBMCs were rinsed and rested in R10 at 37°C and 5%CO_2_ for a minimum of 4 hours. PBMCs were incubated for 6 hours in R10 supplemented with premixed PMA/Ionomycin (Cell Activation Cocktail without Brefeldin A, BioLegend) according to the manufacturer’s instructions. A negative control was also set up using R10 medium without a stimulant for comparison for each sample. All PBMCs were then treated with Brefeldin A (5µg/ml, BioLegend) for the last 4 hours of the culture period for retention of cytokines according to the manufacturer’s instructions.

### Antibodies

The following anti-human antibodies were used for ILC immunophenotyping: lineage-BV510 (CD3, CD14, CD16, CD20, CD56; clone OKT3, M5E2, 3G8, HIB19, 2H7, HCD56), CD127/IL-7Rα-APC (clone A019D5), CD161-PE (clone HP-3G10), CD117/c-kit-BV650 (clone 104D2), CD294/CRTH2-APC-Cy7 (clone BM16), and CD336/NKp44- PE-Cy7 (clone P44-8), all from BioLegend. For intracellular cytokine staining, the following anti-human antibodies were used: IFN-γ-AF488 (clone 4S.B3), IL-13-BV421 (clone JES10-5A2), and IL-22-PerCPCy5.5 (clone 2G12A41), all from BioLegend.

### Staining and Flow Cytometry

PBMCs were washed with 1X PBS then stained with the fixable viability dye, zombie yellow (BioLegend), for 20 minutes in the dark at 4°C. Cells were suspended in cell staining buffer (BioLegend), before labelling with 50µl of the appropriate surface antibody panel for 30 minutes in the dark at 4°C.

For intracellular cytokine staining, cells were fixed using fixation buffer (4% paraformaldehyde, BioLegend), then permeabilized using working strength (1X) intracellular staining perm wash buffer (BioLegend) according to manufacturer’s recommendations. Intracellular cytokine antibodies were prepared in 1X intracellular staining perm wash buffer, and 50µl of the intracellular antibody panel was added and incubated for 30 minutes in the dark at 4°C. After incubation, cells were washed with 1X intracellular staining perm wash buffer twice and finally resuspended in cell staining buffer. Cells were acquired using a BD LSRII flow cytometer.

**Figure 1:**
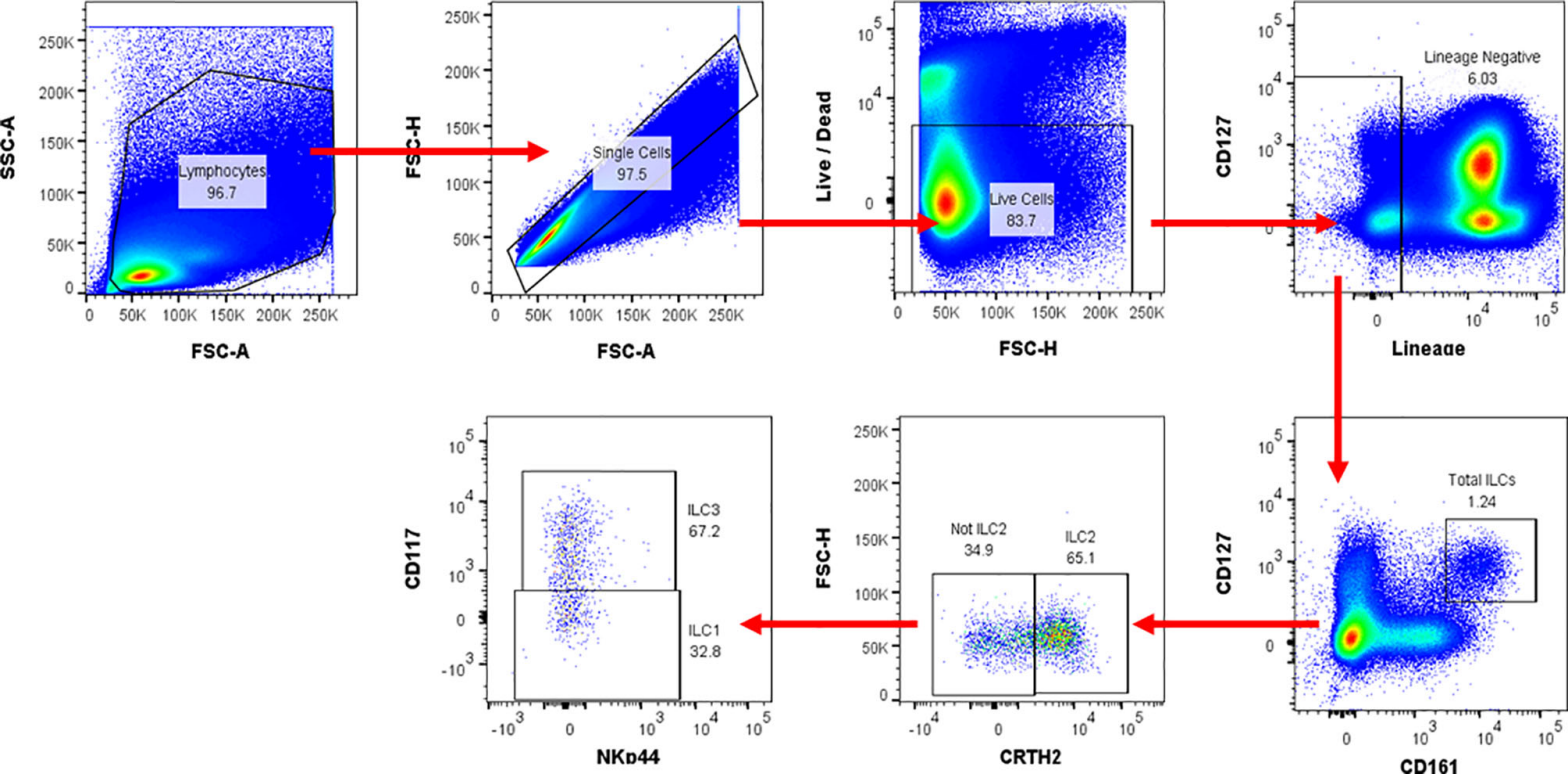
Flow cytometry gating strategy used for ILC identification and cytokine production in human peripheral blood. Representative flow cytometric plots for ILCs in peripheral blood. Numbers in gates (outlined areas) indicate the per cent cell in each. After gating on the time gate, lymphocytes and single cells, distinct ILC populations were defined as: total ILCs (Lin [CD3/CD14/CD16/CD19/CD20/CD56]^−^CD127^+^CD161^+^); ILC1 (Lin^−^CD127^+^CD161^+^CRTH2^-^CD117^−^); ILC2 (Lin^−^CD127^+^CD161^+^CRTH2^+^); ILC3 (Lin^−^CD127^+^CD161^+^CRTH2^+^NKp44^+/−^CD117^+^). The cytokine expression levels for IL-13 and IFN-γ were evaluated using intracellular cytokine staining. FSC-A, Forward scatter-area; FSC-H, Forward scatter-height; SSC-A, Side scatter-area.

All flow cytometry data were analysed using FlowJo version 10.6 for Windows. Gating was standardised and set using Fluorescence Minus One (FMO) controls, and compensation controls to correct for spectral overlap (**Figure 1**). ILC frequencies were expressed as percentages of total lymphocytes. Single live lymphocytes were gated and ILC populations were identified. The distinct populations of ILCs were defined as: total ILCs (Lin^−^CD127^+^CD161^+^); ILC1 (Lin^−^CD127^+^CD161^+^CRTH2^−^CD117^−^), ILC2s (Lin^−^CD127^+^CD161^+^CRTH2^+^), and ILC3s (Lin^−^CD127^+^CD161^+^CRTH2^−^NKp44^+/−^CD117^+^). Gating was performed to determine the percentage expression of IFN-γ by ILC1, IL-13 by ILC2, and IL-22 by ILC3. It’s worth noting that the majority of the NK cells were missed.

### ILC Gating Strategy

#### Statistical Analysis

All statistical analysis was done using GraphPad version 8.3 software. Kruskal Wallis with correction for Dunn’s *post hoc* test was done, and Mann-Whitney U tests for group comparisons. Data were represented as medians and interquartile ranges. Differences were defined as statistically significant with a *P-*value < 0.05 at a 95% confidence level.

## Results

We then assessed frequencies of total ILCs, ILC1, ILC2, ILC3, and the cytokine responses of the ILC sub-sets including IFN-γ, IL-13 and IL-22 by flow cytometry on PBMCs from patients with LTBI and T2DM (n=13), LTBI (n=14), T2DM (n=10) and HC (n=11) (**Table 1**).

**Table 1:** Characteristics of the study population.

	LTBI and T2DM	LTBI	T2DM	HC	*P* Value
	(n = 13)	(n = 14)	(n = 10)	(n =11)	
**Age, years [Median (IQR)]**	51 (40-55.5)	42 (38-49)	55 (41.5-57)	34 (22-40)	0.0045
**Sex, M/F (n)**	5/8	4/10	5/5	3/8	0.662
**Occupation**					0.521
** Businessman/woman**	9 (69%)	7 (50%)	3 (34%)	2 (18%)	
** Skilled labour**	3 (23%)	4 (29%)	4 (44%)	5 (46%)	
** Unskilled labour**	1 (8%)	1 (7%)	1 (11%)	2 (18%)	
** Unemployed**	0 (0%)	2 (14%)	1 (11%)	2 (18%)	
**Religion**					0.526
** Anglican**	3 (23%)	1 (7%)	3 (34%)	2 (18%)	
** Catholic**	2 (15%)	5 (36%)	1 (11%)	2 (18%)	
** Saved/Pentecostal**	1 (8%)	3 (21%)	1 (11%)	0 (0%)	
** Muslim**	7 (54%)	5 (36%)	4 (44%)	7 (64%)	
**Education**					0.386
** Primary**	7 (54%)	6 (43%)	1 (11%)	4 (36%)	
** Secondary**	6 (46%)	8 (57%)	7 (78%)	6 (55%)	
** Tertiary**	0 (0%)	0 (0%)	1 (11%)	1 (9%)	
**Alcohol**					0.875
** Yes**	3 (23%)	3 (21%)	3 (33%)	2 (18%)	
** No**	10 (77%)	11 (79%)	6 (67%)	9 (82%)	
**Smoking**					0.189
** Never smoked**	11 (85%)	14 (100%)	9 (100%)	10 (91%)	
** Used to but stopped**	0 (0%)	0 (0%)	0 (0%)	1 (9%)	
** Still smoking**	2 (15%)	0 (0%)	0 (0%)	0 (0%)	
**BCG Vaccination (n/%)**					0.325
** Yes**	11 (84.6%)	14 (100%)	9 (90%)	10 (91%)	
** No**	2 (15.38%)	0 (0%)	1 (10%)	1 (9%)	
**Fasting blood glucose, mmol/L [Median (IQR)]**	10.7 (8.85-14.6)	6.2 (5.1-7.7)	12.58 (9.325-23.29)	4.9 (4.4-5.5)	<0.0001
**Glycated haemoglobin, % [Median (IQR)]**	8.4 (6.7-10.1)	4.85 (4-5.75)	8.75 (7.3-10.73)	4.3 (4-5.6)	<0.0001

### Phenotypic Analysis of Total ILCs and ILC Subsets

We assessed the frequencies of total ILCs, ILC1, ILC2, and ILC3, and the percentage expression of IFN-γ, IL-13, and IL-22 by gated ILC1, ILC2 and ILC3 cells respectively, using flow cytometry on stored PBMCs from participants with LTBI and T2DM, LTBI, T2DM, and healthy controls.

**Figure 2:**
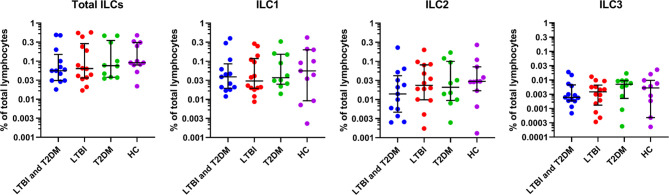
For identification of total ILCs, ILC1, ILC2, ILC3; PBMCs were stimulated and cultured for 6 hours with PMA/ionomycin plus Brefeldin A and stained for surface markers. Size of groups: LTBI and T2DM (n = 13), LTBI (n = 14), T2DM (n = 10), and HC (n = 11). Data represent medians and interquartile ranges. The non-parametric Kruskal-Wallis and Mann-Whitney U tests were used to determine the statistical significance between the medians. Differences were considered statistically significant at P < 0.05 and 95% confidence level. Non-significant P-values were not shown.

The total ILC frequencies were slightly lower in participants with LTBI and T2DM [median (IQR): 0.06 (0.03-0.15)] compared to the healthy controls, but this trend was not statistically supported (**Figure 2**). In addition, there was no significant difference from disease controls (LTBI, and T2DM). The ILC1 frequencies were slightly lower but non-significant in participants with LTBI [median (IQR): 0.03 (0.02- 0.12)] compared to the healthy controls. There were also no observed significant differences with LTBI and T2DM, and T2DM groups. The ILC2 frequencies were slightly lower but non-significant in participants with LTBI and T2DM [median (IQR): 0.01 (0.005-0.04)] compared to the disease (LTBI, and T2DM) and healthy controls. Similarly, the ILC3 frequencies were slightly lower but non-significant in participants with LTBI and T2DM [median (IQR): 0.002 (0.002-0.007)] compared to LTBI and healthy control groups. ILC3 were slightly higher in the T2DM group [median (IQR): 0.007 (0.002-0.010)].

### Functional Analysis of ILC Subsets

**Figure 3:**
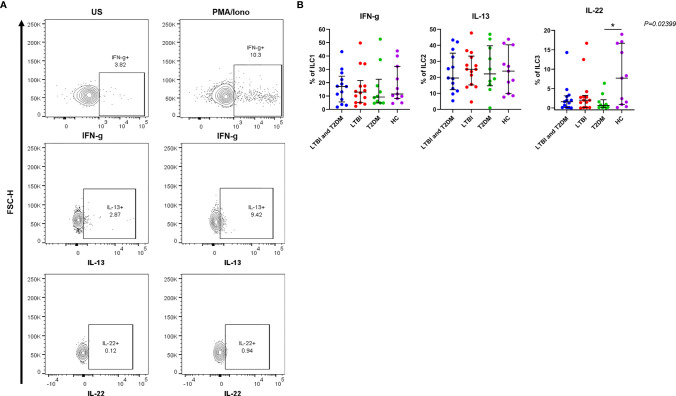
For the production of IFN-γ, IL-13, and IL-22; PBMCs were stimulated and cultured for 6 hours with PMA/ionomycin or unstimulated (US) plus Brefeldin A and stained intracellularly for cytokines. **(A)** Representative flow cytometry plots showing the production of IFN-γ, IL-13 and IL-22 in response to R10 media (unstimulated) and PMA/ionomycin. **(B)** Percentage of ILC1, ILC2 and ILC3 cells expressing IFN-γ, IL-13 and IL-22 respectively. Data represent medians and interquartile ranges. The non-parametric Kruskal-Wallis and Mann-Whitney U tests were performed to determine the statistical significance between the medians. Differences were considered statistically significant at P < 0.05 and 95% confidence level. Non-significant P-values were not shown. Size of groups: LTBI and T2DM (n = 13), LTBI (n = 14), T2DM (N=10), and HC (n = 11) *P-value < 0.05.

IFN-γ production by ILC1 was slightly increased in participants with LTBI and T2DM [median (IQR): 17.1 (5.6-24.9)] and slightly decreased in participants with T2DM [median (IQR): 9.3 (4.8-22.6)] (**Figure 3**). The trend was not different in comparison to LTBI and healthy controls. IL-13 production by ILC2 was slightly increased in participants with LTBI [median (IQR): 25.1 (15.2-33.3)] and slightly decreased in participants with LTBI and T2DM [median (IQR): 19.6 (12.3-35.1)]. The trend was not different in comparison to T2DM and healthy controls. The IL-22 cytokine levels by ILC3 were generally low but production was markedly decreased in participants with T2DM [median (IQR): 0.7 (0.1-2.1)] as compared to healthy controls [median (IQR): 7.7 (0.9-16.7)]. There were no statistical differences in comparison to LTBI and T2DM and LTBI groups.

### Relationship Between ILC Responses and Glycaemic Levels Among T2DM Cases

**Figure 4:**
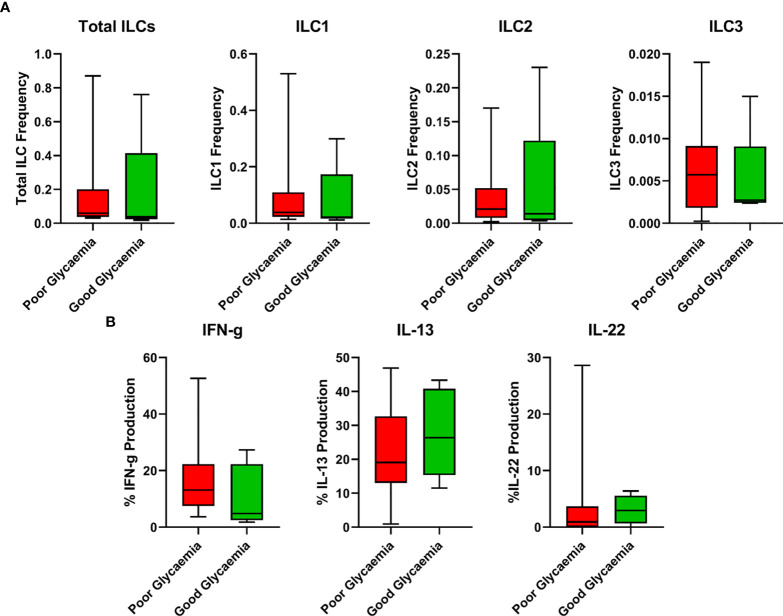
Relationship between ILC responses and diabetes control (Poor and good glycaemic control). **(A)** Responses of total ILCs, ILC1, ILC2, ILC3 in poor and good glycaemic control. **(B)** Responses of IFN-γ, IL-13 and IL-22 in poor and good glycaemic control. Box plots show median values and 25th –75th percentiles from data in each group with maximum and minimum values. Poor glycaemic control was defined as HbA1c levels ≥ 7.0%, and good glycaemic control was defined as HbA1c levels **<**7%. The non-parametric Mann-Whitney U test was performed, and differences were considered statistically significant at P < 0.05 and 95% confidence level. Non-significant P-values were not shown. Size of groups: LTBI and T2DM (n = 13), LTBI (n = 14), T2DM (N=10), and HC (n = 11).

HbA1c is an accurate indicator of the level of diabetic control and increased values reflect poor control. Thus, to examine the relationship between the ILC responses with the degree of diabetic control, we used box plots to assess the association for the frequencies of total ILC, ILC1, ILC2, ILC3, IFN-γ, IL-13, and IL-22, all measured using flow cytometry with HbA1c levels (poor glycaemic control defined as HbA1c ≥7%, and good glycaemic control as HbA1c <7%). We hypothesised that poor glycaemic control further alters ILC responses. As shown in **Figure 4**, poor glycaemic control had slightly higher total ILCs, ILC1, ILC2, ILC3 and IFN-γ responses while good glycaemic control had slightly higher IL-13 and IL-22 responses.

## Discussion

The underlying immunological mechanism for increased tuberculosis in patients with T2DM remains to be more elucidated. Research has shown that T2DM dysregulates immune system components including changes in the number and activation state of immune cell subsets, and altered cytokine levels (40). This study aimed to understand the influence of T2DM on ILC responses in subjects with latent TB infection, with the hypothesis that T2DM alters ILC responses making patients more susceptible to LTBI. ILC responses were variable between the different study groups.

People with T2DM and LTBI had a marginal suppression of immunity as demonstrated by the decreased total ILCs, ILC2 and ILC3 frequencies (**Figure 2**), as well as reduced IFN-γ and IL-13 cytokine production (**Figure 3B**). However, these differences were not statistically supported. IL-22 cytokine production was however greatly decreased among people with T2DM (**Figure 3B**). As anticipated, ILC3 frequencies were slightly higher in the diabetics than in any other study group since these are associated with poor metabolic homeostasis, through the production of IL-17 (41), and on a contrary to alleviate metabolic disorders in diabetes through the production of IL-22 (42). However, in this study, we found very low production of IL-22 by the ILC3s in T2DM. This is similar to a study by Shen et al. (43) that reported IL-22 levels being significantly lower in patients with T2DM compared to those without T2DM. The prototypical type 17 cytokine, IL-22, produced by ILC3s has been shown to play a central role in mediating immunity to both extra and intracellular bacteria, including *Mtb* (44). Ardain et al. (31) also reported that ILC3s mediate early protective immunity against TB infection, and IL-22 production by ILC3s was critical for early innate immunity and granuloma formation. Another study reported IL-22 production by two ILC3 subsets, lymphoid tissue inducers (LTi) and natural cytotoxicity receptor (NCR)^+^ ILC3s, to be significantly decreased in *Mtb*-infected mice with T2DM than in *Mtb*-infected mice without T2DM (45). The authors further demonstrated that Mtb-infected T2DM mice had prolonged survival following recombinant IL-22 treatment or adoptive ILC3 transfer (45). It is paradoxical to have low IL-22 cytokine responses despite high ILC3 expression. This is probably because ILC3s weren’t activated in response to any of the disease states, or the type 17 cytokine, IL-17A was instead produced, but it was not measured. It is also probable that T2DM inhibited IL-22 cytokine production, mechanisms of which were not assessed in this study.

Also of major importance is IFN-γ, the functions of which are well documented in both mouse models and human infections (46, 47). IFN-γ is a key Th-1 signature cytokine that is up-regulated by *Mtb* infection, critical for tuberculosis control (48), however, this may not translate into immune correlates of protection against TB (49). Ardain et al. (31) reported that blood ILC1s were depleted in participants with TB compared to controls, and these rebounded after treatment, indicating that TB influences blood ILC1 responses. They further demonstrated that Lung ILC1s do not rapidly accumulate in the lung following TB infection and that *Ifng*−/− mice maintained *Mtb* control. Indeed, Sakai et al. (50) earlier showed that IFN-γ responses are only approximately 30% of the CD4 T cell cumulative *Mtb* control, but over 80% in the spleen. Taken together, the data shows that the role of ILC1-derived IFN-γ may be in the blood and extra-pulmonary sites. In T2DM, blood and adipose tissues had elevated ILC1s and IFN-γ compared to normal individuals (36, 37). The authors further demonstrated that elevated ILC1^+^IFN-γ^+^ cells resulted in an increased risk of T2DM (36). This shows that TB and T2DM independently modulate ILC responses. Whether this applies to LTBI-T2DM comorbidity remains to be assessed. In this study, patients with LTBI and T2DM had a slightly higher production of IFN-γ. This finding may appear to be paradoxical because patients with T2DM are more susceptible to LTBI. A possible explanation is that type 2 diabetic patients show changes in the downstream signal transduction of key Th1 and innate immune response cytokines, perhaps because of increased advanced glycation end products that bind and modify protein function (51). This suppresses downstream responses important in eliminating *Mtb*, despite high levels of protective cytokines. This possibly results from the failure of cytokines to induce negative feedback mechanisms, that normally control their expression or an increased half-life of cytokines because of delayed proteolysis resulting from binding of advanced glycation end products (52). Therefore, probable that T2DM impairs the immune response in LTBI, resulting in insufficient production of protective ILC1 responses, potentially increasing susceptibility to active TB disease. Our data shows reduced ILC1 among individuals with LTBI only. This could be as a result of the immune-modulating effects of *Mtb* including preventing activation of pathogen destruction systems (53), and blocking the presentation of antigens on class II MHC by action on endosomal sorting complexes required for transport (ESCRT) of its EsxG·EsxH protein (53).

Obesity induces low-grade chronic inflammation in the white adipose tissue (WAT) increasing the risk of metabolic disorders (54, 55). Type 2 responses are associated with the recruitment of alternatively activated macrophages (AAMs) that promote the development of functional beige fat (56). ILC2s have been identified to regulate eosinophil and AAMs recruitment through the production of IL-5 and IL-13 (18), and to limit obesity in mice and human WAT (19, 57). In these studies, obesity in mice and humans is associated with decreased ILC2s (19, 57). Ardain et al. (31) demonstrated that blood ILC2s to be involved in the pathogenesis of TB. ILC2s are depleted during TB infection despite being activated and accumulate in the lung following TB infection (31). This remains to be assessed in a metabolic disorder like obese associated-T2DM, and its effects on LTBI in humans. In our study, we demonstrate that participants with LTBI and T2DM had slightly lower ILC2 levels (**Figure 2**), and IL-13 production compared to other groups (**Figure 3B**). Indeed, T2DM participants had slightly lower ILC2 levels and IL-13 production. This is consistent with studies of adaptive immunity that demonstrate a decrease in Th2 cellular responses in participants with LTBI-type 2DM compared to LTBI without T2DM. Hence T2DM may be associated with a general decrease in ILC2 responses. ILC2 production of type 2 cytokines that results in AAMs recruitment (19, 57) may be considered detrimental in controlling *Mtb.* This may be contradictory as Sudo et al. (58) reported that ILC2s produce GM-CSF, a cytokine known to activate macrophages and control *Mtb* infection (59). This may explain the slight increase in the ILC2 and IL-13 levels in the LBTI group.

Immune responses are known to be altered in T2DM, with hyperactivation of T-cells as a significant feature (60). Poor glycaemic control is associated with poor infection control (61), and elevated inflammatory activity (62). ILCs have been implicated to be critically involved in the pathogenesis of chronic inflammatory diseases including obesity and T2DM (35–38), Crohn’s disease (63), multiple sclerosis (64) and psoriasis (65). However, a direct relationship between ILC responses and T2DM remains to be elucidated. Studies by Liu et al. (37) and Wang et al. (36) reported a positive correlation between adipose and circulating ILC1s with HbA1c and fasting plasma glucose (FBG). In this study, we report that the association between HbA1c levels and ILC immune responses indicate a general minimal alteration of ILC immune responses in poor and good glycaemic control (**Figure 4**). Poor glycaemic control had slightly higher total ILCs, ILC1, ILC2, ILC3 and IFN-γ responses but slightly lower IL-13 and IL-22 responses. The further depressed IL-22 with poor glycaemic control suggests more immune suppression in T2DM and plausible susceptibility to TB infection.

Humans show significant sex differences in the incidence and severity of respiratory diseases, including asthma and virus infection. Sex hormones (oestrogens and androgens) contribute to the female sex bias in type 2 inflammation associated with respiratory diseases, consistent with recent reports that female lungs harbour greater numbers of GATA-3–dependent group 2 innate lymphoid cells (ILC2s) (66–68). In the current study, we analysed ILC subsets and cytokine responses based on sex differences to determine whether there are any ILC response differences. There were no distinct differences in ILC responses based on sex (**Supplementary Figures 1A, B**). Notably, no changes in effector functions were found between male and female ILC2s when stimulated with PMA/ionomycin, indicating a selective deficit in IL2R- or ST-2-signaling pathways imprinted by androgen signalling (66). In this study, PBMCs were stimulated with PMA/Ionomycin, and this could explain the observations found.

The frequency and functional capacity of ILCs over the human lifespan has been demonstrated. Darboe et al. (69) assessed the numbers and frequencies of ILC subsets in peripheral blood in a group of Gambians aged 5 to 73. The authors reported that ILC1 frequencies were stable whereas ILC3 absolute numbers and frequency decreased steadily throughout life, as ILC2 absolute numbers and frequency decreased from childhood until around the age of 30 (69). In our study, we report that the frequency of ILC subsets declined throughout life, with total ILC and ILC2 frequencies declining quite markedly (**Supplementary Figures 2A, B**). Though not markedly, the decline of ILC1 frequency with age is in agreement with a study that reported a decline of frequency and absolute counts of ILC-1 like cells with gestational age (70). The authors further show that ILC2 and ILC3 frequencies declined markedly (70). This partially agrees with this study that reported slight declines of ILC2 and ILC3 frequencies respectively. Functionally, IFN-*γ* markedly declined whereas IL-13 and IL-22 slightly declined with age, reflecting declining functional attributes of ILC subsets with age. This may be attributable to functional redundancy of CD4+ T cells and the ILC subset counterparts (71). The divergent results in these studies may reflect differences in study design.

There is evidence of plasticity (ILC3 to ILC1, and ILC2 to ILC1) demonstrated in both humans and mice, producing IFN-γ in both conversions. However, ILC3 and ILC2 plasticity requires inflammatory cytokines such as IL-23, IL-15, IL-12 and IL-1β. ILC3 plasticity has been demonstrated in Crohn’s disease (72), while ILC2 plasticity in COPD (73) and Crohn’s disease (74). T2DM being a chronic inflammatory disease could change the cytokine microenvironment affecting the conversions. This has not been fully demonstrated in T2DM. Indeed, corral et al. (75), (unpublished) demonstrated that ILC1-like-IFN-γ producing cells that expressed little to no classical ILC2 markers emerged during *Mtb* infection. The authors further illustrated that metabolic environment inhibits ILC2 while augmenting ILC1-like- IFN-γ producing cells (75). The current study did not assess for the origin of ILC1 and IFN-γ production, therefore did not evaluate ILC2 and ILC3 plasticity.

This study suffered limitations of being descriptive, having a small sample size, and measuring only percentages of ILC sub-sets as opposed to absolute cell numbers. Also, the study did not collect data on confounding variables such as helminth infection or allergy status that may affect the ILC responses. This study was able to show the effect of T2DM on ILC immune responses in individuals with latent TB infection. It would be interesting to study the impact of T2DM on ILC immune responses in individuals with active TB, and the PTB-T2DM impact on other pathogens.

## Conclusion

Our data imply that observed ILC sub-set immune alterations are driven by chronic hyperglycaemia. It also supports the growing body of evidence that confirms that immunodeficiency is an integral part of T2DM and that this compromise could have serious consequences in the face of intracellular pathogens that require type-1 associated cytokines for resistance. This study provides an impetus to perform longitudinal studies examining the role of T2DM related immunosuppression in the progression from latent infection to active tuberculosis in the face of innate lymphoid cells. It is also a gap to further assess the transcription factors and genes that are upregulated and downregulated by innate lymphoid cells in LTBI-T2DM patients, and to study lung tissues and determine whether the innate lymphoid cells seen are either tissue-resident or circulating. Future studies could further look at the dysregulation of the innate lymphoid cells in patients having active tuberculosis and type 2 diabetes mellitus, and assess the effects on treatment response of both disease entities.

## Data Availability Statement

The original contributions presented in the study are included in the article/**Supplementary Material**. Further inquiries can be directed to the corresponding author.

## Ethics Statement 

The studies involving human participants were reviewed and approved by School of Biomedical Sciences Research and Ethics Committee College of Health Sciences, Makerere University. The patients/participants provided their written informed consent to participate in this study.

## Author Contributions 

PS, MN, CM, and BM conducted the experiments. PS, RS, DD, SC, and IB designed the research study. PS, MN, ME, MH, DD, SC, and IB analysed data. PS, MN, RS, ME, CM, BM, MH, DD, SC, and IB all discussed results, wrote and/or reviewed the manuscript. All authors contributed to the article and approved the submitted version.

## Funding

The Makerere University/UVRI Infection and Immunity Research Training Programme (MUII)-plus is funded by the Wellcome Trust (Grant number 084344) through the DELTAS Africa Initiative, under the African Academy of Sciences Alliance for Accelerating Excellence in Science in Africa (AESA). The funding organisations have not contributed to any of the findings in this publication.

## Conflict of Interest

The authors declare that the research was conducted in the absence of any commercial or financial relationships that could be construed as a potential conflict of interest.

## Publisher’s Note

All claims expressed in this article are solely those of the authors and do not necessarily represent those of their affiliated organizations, or those of the publisher, the editors and the reviewers. Any product that may be evaluated in this article, or claim that may be made by its manufacturer, is not guaranteed or endorsed by the publisher.
