# Supplementary material for: Type 2 Diabetes Mellitus and Latent Tuberculosis Infection Moderately Influence Innate Lymphoid Cell Immune Responses in Uganda
Source: Front Immunol. 2021 Aug 27;12:716819. doi: 10.3389/fimmu.2021.716819 (PMC8432960; doi:10.3389/fimmu.2021.716819)
Supplement: Supplementary file 1 [file DataSheet_1.docx]

**
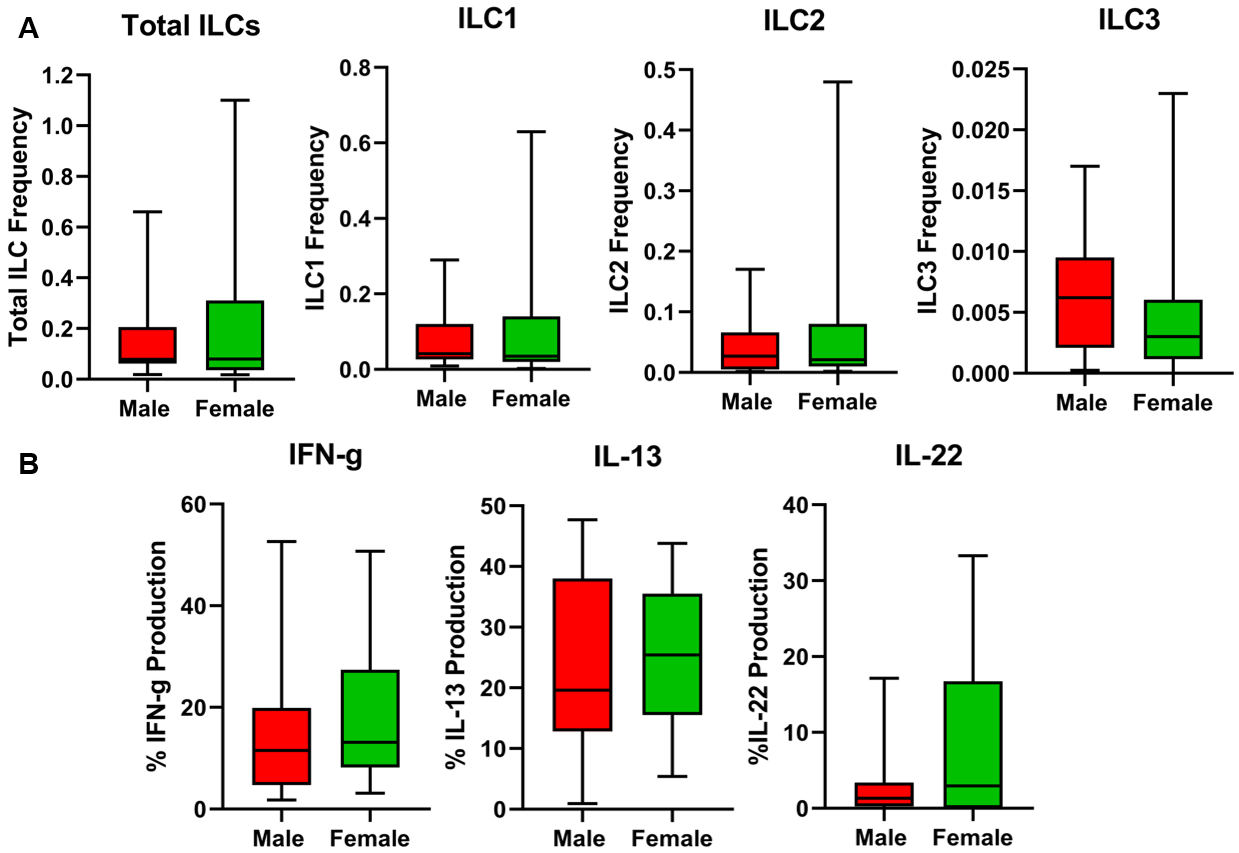
**

**SUPPLEMENTARY FIGURE 1 |** Analysis of ILC responses based on sex differences between male and female participants. Box plots show median values, 25^th^–75^th^ percentiles from data in each group with maximum and minimum values. Differences were considered statistically significant at P < 0.05 and 95% confidence level. Non-significant P-values were not shown. Size of groups: LTBI and T2DM (n = 13), LTBI (n = 14), T2DM (N=10), and HC (n = 11).

**
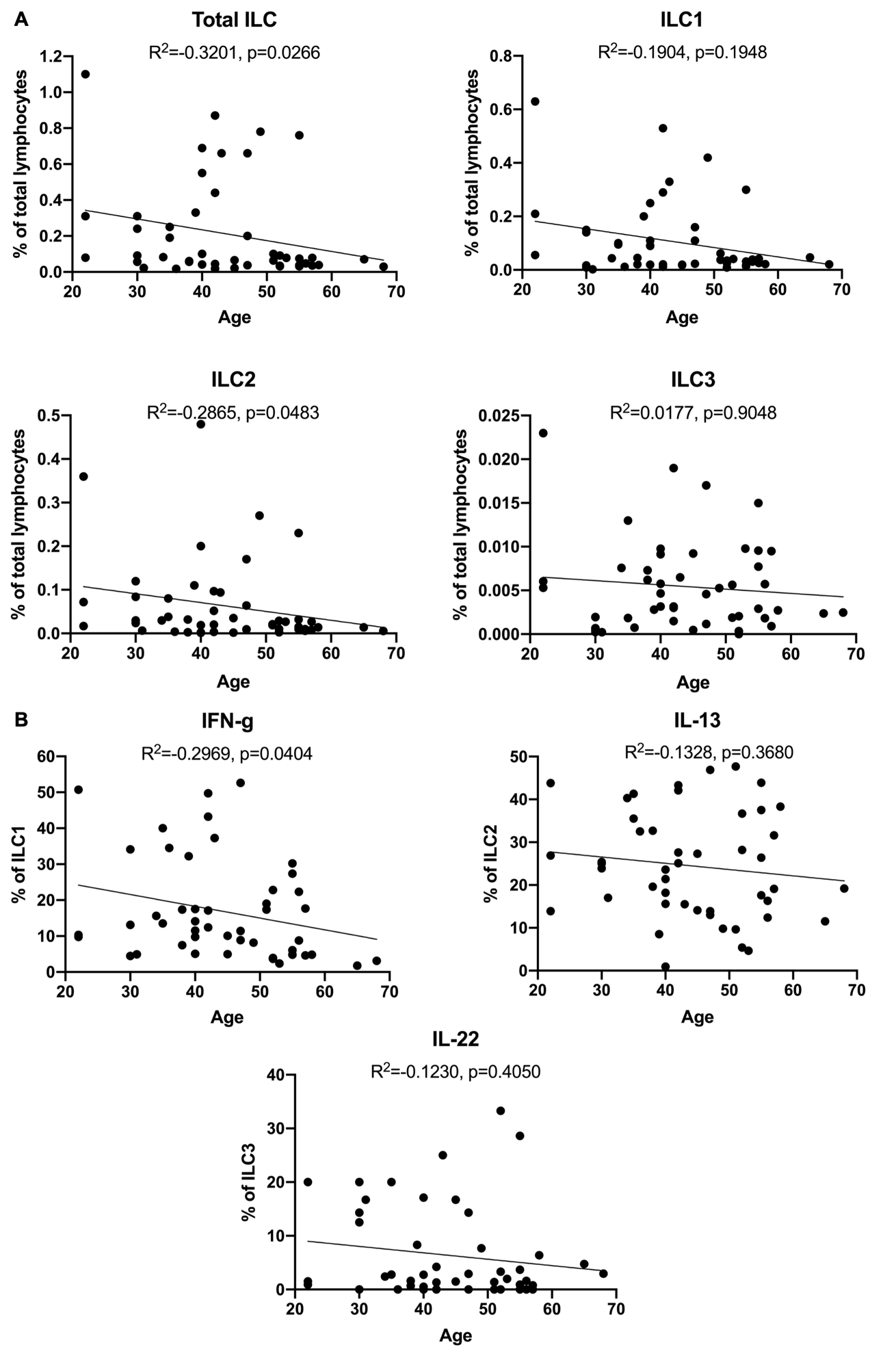
**

**SUPPLEMENTARY FIGURE 2 |** Changes in frequency of ILC subsets and percentage cytokine production over the age of 22 to 68 years. **(A)** Frequencies of total ILC, ILC1, ILC2 and ILC3. **(B)** ILC functional responses to IFN-γ, IL-13 and IL-22 production. Data are represented as scatter plots, with each dot representing a single individual. P-values were calculated by Spearman rank correlation.

**SUPPLEMENTARY TABLE 1 |** Representative medians and interquartile ranges for total ILC, ILC1, ILC2, ILC3, IFN-γ, IL-13 and IL-22 in the study groups. Data are represented as medians and interquartile ranges.

| **Parameter** | **LTBI and T2DM**  **(*n = 13*)** | **LTBI**  **(*n = 14*)** | **T2DM**  **(*n = 10*)** | **HC**  **(*n =11*)** | ***P-*Value** |
| --- | --- | --- | --- | --- | --- |
| Total ILCs | 0.056 (0.031-0.150) | 0.064 (0.036-0.290) | 0.076 (0.038-0.349) | 0.091 (0.079-0.310) | 0.467 |
| ILC1 | 0.039 (0.020-0.086) | 0.031 (0.020-0.118) | 0.037 (0.025-0.153) | 0.056 (0.009-0.200) | 0.890 |
| ILC2 | 0.014 (0.005-0.042) | 0.024 (0.010-0.081) | 0.021 (0.009-0.101) | 0.030 (0.017-0.072) | 0.561 |
| ILC3 | 0.002 (0.002-0.007) | 0.004 (0.001-0.007) | 0.007 (0.002-0.010) | 0.005 (0.0005-0.010) | 0.689 |
| IFN-γ | 17.100 (5.570-24.850) | 12.950 (5.043-21.650) | 9.265 (4.825-22.600) | 11.500 (8.180-32.200) | 0.930 |
| IL-13 | 19.600 (12.250-35.100) | 25.05 (15.230-33.250) | 22.15 (14.730-39.700) | 23.900 (9.800-40.300) | 0.976 |
| IL-22 | 1.610 (0.100-3.135) | 1.995 (0.100-3.233) | 0.650 (0.100-2.120) | 7.690 (0.900-16.70) | 0.111 |
